# Effects of a Physical Activity Program on Markers of Endothelial Dysfunction, Oxidative Stress, and Metabolic Status in Adolescents with Metabolic Syndrome

**DOI:** 10.5402/2012/970629

**Published:** 2012-07-25

**Authors:** Eneida Camarillo-Romero, Ma Victoria Dominguez-Garcia, Araceli Amaya-Chavez, Maria del Socorro Camarillo-Romero, Juan Talavera-Piña, Gerardo Huitron-Bravo, Abraham Majluf-Cruz

**Affiliations:** ^1^Centro de Investigación en Ciencias Médicas, Universidad Autónoma del Estado de México, 50130 Toluca Estado de México, Mexico; ^2^Facultad de Química, Universidad Autónoma del Estado de México, 50120 Toluca Estado de México, Mexico; ^3^Centro de Adiestramiento en Investigación Clínica, Coordinación de Investigación en Salud, Instituto Mexicano del Seguro Social, México, DF, Mexico; ^4^Unidad de Investigación Médica en Trombosis, Hemostasia y Aterogénesis, Instituto Mexicano del Seguro Social, Apartado Postal 12-1100, México, DF, Mexico

## Abstract

The metabolic syndrome (MetS) is a precursor of diabetes. Physical activity (PA) improves endothelial dysfunction and may benefit patients with MetS. *Aims*. To evaluate the effect of a physical activity (PA) program on markers of endothelial dysfunction and oxidative stress in adolescents with (MetS). *Methods*. We carried out a cohort study of 38 adolescents with and without MetS (18 females and 20 males). All participants completed a 3-month PA program. All variables of the MetS as well as markers of endothelial dysfunction and oxidative stress tests were evaluated. *Results*. Females with and without MetS showed significant differences for almost all components of the MetS, whereas males were significantly different in half of the components. After the PA program, components of the MetS were not different from baseline values except for HDL-C levels. Some baseline endothelial dysfunction markers were significantly different among adolescents with and without MetS; however, after the PA program, most of these markers significantly improved in subjects with and without MetS. *Conclusion*. PA improves the markers of endothelial dysfunction in adolescents with MetS although other changes in the components of the MetS were not observed. Perhaps the benefits of PA on all components of MetS would appear after a PA program with a longer duration.

## 1. Introduction

Metabolic syndrome (MetS) is a cluster of several atherothrombotic risk factors and is considered to be a precursor of atherothrombotic diseases and diabetes mellitus [1]. Several criteria have been proposed for diagnosis of MetS. The importance of establishing the diagnosis of MetS is that these individuals have a 1.5- to 2.0-fold increase in risk for atherothrombotic events [2], a predictive effect that is similar to that of the Framingham Score [3].

The prevalence of MetS in adolescents according to the Adult Treatment Panel III (ATP-III) definition varies significantly. For example, the prevalence of MetS is higher in Mexico than in the US (19.6% versus 8.6%, resp.) [4, 5]. However, when the criteria of the International Diabetes Federation (IDF) are used, the prevalence of MetS in Mexico and the U.S. does not differ greatly (8.2% versus 4.5%, resp.) [6, 7]. In overweight or obese adolescents, the incidence of MetS increases to 38% and 50%, respectively [6, 8].

Children older than 10 years with overweight or obesity have a high risk for the development of dyslipidemia, arterial hypertension, abnormalities in carbohydrate metabolism, and prothrombotic and proinflammatory states [9]. One necropsy study in adolescents and young adults demonstrated that all the above-mentioned atherogenic conditions begin very early in life [10]. Obese children without other risk factors have markers of endothelial cell (EC) dysfunction [11, 12]. In obese adolescents, markers of EC dysfunction such as high-sensitivity C-reactive protein (hsCRP) and soluble cell adhesion molecules (CAMs) have been described in association with the early stages of the atherosclerotic process. These facts strongly suggest that endothelial dysfunction is one of the seminal pathophysiological abnormalities in atherosclerosis and that its detection may possibly be used as an early marker of atherothrombotic disease [11].

Several studies have fully demonstrated the beneficial effects of physical activity (PA) in different groups in healthy controls and in patients with several pathological states [13–18], including MetS [19]. This body of evidence also shows that, in adults, PA induces beneficial local and systemic changes in the fibrinolytic system as well as in the vascular metabolism and muscles that may protect against atherothrombotic events.

Due to the very well-known beneficial effects of PA in adult patients with MetS, the aim of this study was to evaluate the effects of a PA program on metabolic parameters and markers of endothelial cell dysfunction and oxidative stress in adolescents with MetS.

## 2. Materials and Methods

### 2.1. Study Design

**Figure 1:**
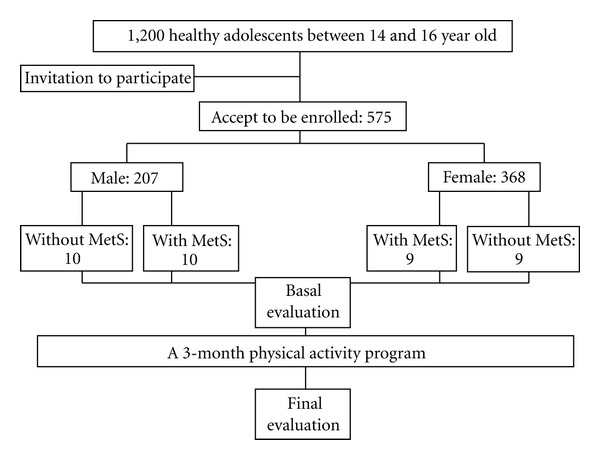
Study flow chart. Thirty-eight nonsmoking adolescents with and without MetS were enrolled. MetS: metabolic syndrome.

A cohort study including 1200 healthy adolescents between 14 and 16 years of age was carried out between February 2011 and May 2011. A search for MetS was performed. Finally, 575 adolescents accepted to participate (207 males and 368 females) (Figure 1). According to the IDF criteria, 47 participants (8.2%) had MetS. Because waist circumference was higher than the 90th percentile, which was above the cut-off level according to the IDF definition, we used the criteria for adults (males ≥90 cm; females ≥80 cm). Measurement of waist circumference was standardized and strictly evaluated according to the recommendation of the World Health Organization (WHO) [20]. Blood pressure was evaluated twice with a mercury sphygmomanometer with the subjects in a seated position after a resting period [21].

### 2.2. Interventions

For this study we included 38 age- and gender-matched adolescents (18 females and 20 males): 19 with MetS and 19 without MetS. Exclusion criteria included smoking and presence of infectious disease at the time of evaluation or during the 15 days prior to enrollment or taking anti-inflammatory or antihistamine drugs during the same time period. Alcohol intake during the study was highly discouraged. Pregnancy was always considered as an exclusion criterion. Both groups participated in a 3-month PA program. The program was designed to exercise 45 min/day for 5 days/week with an intermediate intensity (cardiac rate = 60% to 70%, 3 to 6 METs/min) as monitored with a heart rate monitor. This program was designed and supervised by an international certificated instructor. Importantly, although some dietary advice was offered, no specific changes in dietary habits or meal characteristics were indicated to the study participants.

Before and after the PA program, fasting blood samples (10 mL) were obtained using a vacuum system. Blood was drawn in vacuum tubes containing either sodium citrate 3.8% (vol : vol, 1 : 9), heparin, EDTA, and in another tube without anticoagulant. All tubes were centrifuged for 10 min at 500 g in order to obtain platelet-poor plasma and serum. Triglycerides (TGs), high-density lipoprotein cholesterol (HDL-C), and fasting glucose measurements were performed on the same day of the collection using an enzymatic assay (RX Daytona Randox Laboratories Ltd., County Antrim, UK). We measured soluble vascular cell adhesion molecule (VCAM-1) and P-selectin (Invitrogen, Carlsbad, CA, USA). Also, plasma von Willebrand factor (vWF) was evaluated using a commercially available kit (Asserachrome vWF : Ag, Stago, Asnieres, France). hsCRP was assayed using a commercially available kit (CRPH High Sensitivity C-Reactive Protein Reagent; Beckman Coulter; Mervue, Galway, Ireland) in an automated equipment system (IMMAGER Immunochemistry System; Beckman Coulter). Several fibrinolytic variables were evaluated, namely, plasminogen activator inhibitor (PAI-1) (Asserachom PAI-1, Stago), tissue plasminogen activator (tPA) (Asserachrom tPA, Stago), factor VIII (FVIII) (STA Factor VIII, Stago), and fibrinogen (Fibrinogen Reagent, Stago). Hemoglobin was evaluated in the blood collected in EDTA tubes using an AcT Diff Hematology Analyzer (Beckman Coulter, Fullerton, CA, USA). Heparinized blood was used for assays evaluating oxidative stress. Lipoperoxidation, enzymatic activity of superoxide dismutase, catalase activity, and glutathione peroxidase activity were evaluated according to previously described and validated methods [22–25].

### 2.3. Statistical Analysis

We described baseline value and values after the PA program (mean and standard deviation) of the components of the MetS as well as markers of inflammation, EC dysfunction, and oxidative stress. Differences of these variables between cases and controls were established using Student's *t*-test. We then described the mean and standard deviation of the same variables in males and females after application of the PA program. Before and after results were compared using a paired *t*-test, significant differences were considered when *P* < 0.05. All statistical analyses were performed using a Statistical Package for the Social Sciences (SPSS) software (v.16; SPSS Inc., Chicago, IL, USA).

### 2.4. Ethical Considerations

Before entering the study, all participants and their parents were informed about the characteristics and objectives of the investigation. Signed informed consent was obtained from the parents and from each adolescent. The study was conducted according to all the principles of the Declaration of Helsinki. The protocol was approved by the ethics committee of our research center.

## 3. Results

As previously mentioned, 19 adolescents (9 females and 10 males) with MetS were included and compared with a paired control group without MetS. Baseline general characteristics of the adolescents in both groups are shown in Table 1. Females with and without MetS showed differences for all the components of the MetS, except for diastolic blood pressure. On the contrary, males with and without MetS were significantly different in only half of the components of the MetS. After completing the PA program, most of the components of the MetS were not significantly different from baseline levels in adolescent females and males, with and without MetS (Table 2). Only levels of HDL-C significantly increased after the PA program in females and males in both groups. Also, in females and males with MetS, a significant reduction in fasting glucose levels was observed after the PA program.

Table 3 shows baseline levels of markers of EC dysfunction. As shown, only some variables were different in males and females with and without MetS. However, when we compared the data in regard to the same markers after completing the PA program, most inflammatory and EC dysfunction markers decreased in all study participants with and without MetS (Table 4). As depicted in Table 4, results of the analysis of oxidative stress were contradictory.

## 4. Discussion

### 4.1. Metabolic Syndrome and Endothelial Cell Dysfunction

 MetS is caused by a combination of genetic factors, poor dietary habits, and sedentary lifestyle. It is a major risk factor for atherothrombotic diseases [26]. According to some cohorts of adolescents from several countries, the prevalence of MetS ranges from 8.2 to 12.6% [6].

Upon appropriate stimulation with inflammatory mediators the endothelium may become dysfunctional. Endothelial dysfunction is defined as impairment in any of the normal functions of the ECs leading to vasoconstriction, thrombosis, upregulation of CAMs, increased cytokine and chemokine secretion, and leukocyte adherence, among others. EC dysfunction is regarded as a precursor for atherogenesis, and it is associated with a high risk of atherothrombotic events [27, 28]. Therefore, EC dysfunction seems to be the common link between risk factors and atherosclerosis. Thus, restoration of normal EC function is a highly desirable therapeutic goal.

### 4.2. Endothelial Cell Dysfunction and Atherosclerosis

ECs have other functions in addition to the regulation of vascular tone. Today it is possible to measure plasma or serum constituents as alternative markers of endothelial function. For example, CAMs are expressed on the surface of ECs in response to endothelial dysfunction, and their circulating levels are considered as surrogate markers of dysfunction, or damage [29]. Elevated soluble CAMs are found in patients with vascular risk factors and early or established atherosclerosis and may predict future atherothrombotic events [29]. On the other hand, thrombus is central to the progression of atherosclerosis. Several blood coagulation factors are proposed as markers of EC dysfunction, namely, increased vWF, fibrinogen, tPA, and PAI-1 [30]. Also, hsCRP are a predictor of atherothrombotic disease [31]. Because atherosclerosis is an inflammatory process, markers of inflammation such as hsCRP is an indirect marker of EC dysfunction and may be useful for global assessment of atherothrombotic risk, especially among individuals without known atherothrombotic disease [29]. Finally, because oxidoreduction is important for the regulation of EC function, oxidative stress contributes to EC dysfunction and may be central in atherogenesis due to increased lipoperoxidation and free radical production [32]. Reactive oxygen species may interact with proteins, lipids, and DNA, and their excessive production is implicated in atherogenesis [33].

### 4.3. Effects of PA on Markers of Endothelial Cell Dysfunction and Metabolic Variables

We attempted to determine the pattern of markers of EC dysfunction before and after a PA program in adolescents with and without MetS. We measured several markers of endothelial dysfunction which are widely used and accepted in the literature [12, 15, 17–19, 34–36]. As expected, we found significant differences for the components of the MetS between male and female adolescents with and without this syndrome. These differences were found for most of the components, a fact that suggests that, indeed, we had two significantly different populations from a metabolic perspective. Although there was a trend towards lower levels of most of the markers of EC dysfunction, results were not significant between adolescents with MetS versus those without MetS. An explanation for this finding may be that some adolescents of the control group had one or more of the components of the MetS, although they did not fulfill the criteria to be considered carriers of the syndrome. However, as previously shown, some of the clinical criteria of the MetS may directly induce EC dysfunction. As a consequence, some control individuals may have markers of EC dysfunction, although they did not fulfill the criteria to be considered as carriers of MetS, a fact that impeded us to find significant differences between groups.

Our most important findings appeared after the PA program. We found significant changes in HDL-C levels in all groups; however, no changes were found for TG as previously shown [37]. An explanation for this finding may be that we did not include a dietary intervention; thus, some adolescents may increase their intake of carbohydrates. Glucose improved only in the groups of adolescents with MetS, and blood pressure did not change and may be explained because this was the most infrequent component of the MetS in our study population. Regarding analysis of markers of EC dysfunction, we found a significant effect in most of these markers after the completion of the PA program. In terms of inflammatory markers, only hsCRP was not modified, and a similar nonbeneficial effect was observed for the majority of the oxidative stress tests. Only lipoperoxidation levels were significantly lower in male and female adolescents with MetS. On the contrary, the most important findings in our research derived from the changes in the EC dysfunction markers in adolescents with MetS as well as in the controls without MetS, as previously described in healthy populations [13]. Except for vWF levels, almost all markers of EC dysfunction significantly improved after exercising, even for P-selectin levels in male adolescents with MetS. These did not reach significant differences but there is a clear trend towards improvement after PA.

The effects observed after the PA program are not that surprising. Indeed, many atherothrombotic risk factors associated with MetS can be reduced or eliminated through weight control, diet, and, most importantly, regular exercise [38]. PA induces beneficial effects in patients with chronic heart failure, coronary artery disease, arterial hypertension, and hypercholesterolemia [14, 39]. Moderate-vigorous daily PA prevents both the incidence of chronic diseases and premature death [40]. The positive effects of PA include increased expression of nitric oxide synthase and EC-derived superoxide dismutase, improvement in vascular structure and local and systemic fibrinolysis, and improvement of coronary EC function [41–43]. Regular PA helps in achieving appropriate weight loss and improves insulin sensitivity and EC function in insulin-resistant individuals [44]. Also, there is a significant association between PA and a reduction of inflammatory cytokine levels, an effect that is associated with improvement of the EC function [45]. Finally, it has been shown that PA improves EC function by increasing the bioavailability of nitric oxide although its beneficial effect is lost within weeks after the end of training [46]. This body of evidence suggests that EC function is modulated by the extent of daily PA.

### 4.4. Study Limitations

In this study we found a beneficial effect of PA on markers of EC dysfunction, namely, CAMs and blood coagulation markers; however, we could not demonstrate a positive consequence for other variables such as hsCRP and oxidative stress tests. We can hypothesize that this lack of effect may be due to a PA program of insufficient length to induce the desired changes. Perhaps each marker of EC dysfunction requires a specific time in order to be reversed with PA, and such timing possibly was not fully achieved in our study for all variables analyzed.

Most studies on EC dysfunction have been performed in adults with specific pathological states and only a few have addressed children and adolescents [47]. In adolescents, a 45 min/day moderate PA significantly reduces the risk of MetS, prevents overweight, effectively reduces weight, and improves the oxidative and cytokine profile [48, 49]. However, most of these studies are cross-sectional, and, to our knowledge, none of the previous studies has shown the beneficial effects of PA on markers of EC dysfunction.

## 5. Conclusions

We have shown that a PA program effectively reduces the levels of several markers of EC dysfunction in adolescents with MetS, although these beneficial changes are not associated with the expected changes in the clinical components of the MetS. Perhaps these benefits on EC dysfunction markers may be increased or extended either to inflammation markers, to oxidative stress tests, or to the clinical components of MetS with a PA program beyond 3 months.

## Figures and Tables

**Table 1:** Baseline status of the components of the MetS in adolescents.

	Women (*n* = 18)			Men (*n* = 20)		
Component	*n*/% (mean ± SD)			*n*/% (mean ± SD)		
	With MetS	Without MetS	P*	With MetS	Without MetS	P*
WC	9/100 (90 ± 10)	0/0 (68 ± 6)	<0.001	10/100 (98 ± 8)	3/30 (81 ± 7)	<0.001
SBP	0/0 (111 ± 10)	0/0 (97 ± 3)	0.008	1/10 (118 ± 10)	0/0 (116 ± 6)	0.568
DBP	2/22 (76 ± 8)	1/11 (69 ± 14)	0.269	1/10 (78 ± 9)	0/0 (68 ± 9)	0.023
HDL-C	6/66 (35 ± 6)	3/33 (64 ± 19)	0.001	8/80 (38 ± 18)	8/80 (55 ± 27)	0.150
TG	5/55 (115 ± 54)	0/9 (68 ± 17)	0.036	6/60 (113 ± 63)	1/10 (69 ± 33)	0.083
Glucose	3/33 (101 ± 9)	0/9 (83 ± 14)	0.009	6/60 (102 ± 8)	2/20 (83 ± 12)	0.002

WC: waist circumference (cm); SBP: systolic blood pressure (mmHg); DBP: diastolic blood pressure (mmHg); HDL-C: high-density cholesterol (mg/dL); TG: triglycerides (mg/dL); glucose (mg/dL). ^∗^Sudent's *t*-test.

**Table 2:** Effects of PA on the components of the MetS in adolescents.

	Women (*n* = 18)						Men (*n* = 20)					
	Mean (SD)						Mean (SD)					
	With MetS (*n* = 9)			Without MetS (*n* = 9)			With MetS (*n* = 10)			Without MetS (*n* = 10)		
	Basal	PPA	P*	Basal	PPA	P*	Basal	PPA	P*	Basal	PPA	P*
WC	90 (10)	87 (10)	0.203	68 (6)	72 (5)	0.026	98 (8)	89 (9)	<0.001	81 (7)	76 (8)	0.251
SBP	111 (10)	105 (10)	0.171	97 (3)	98 (5)	0.691	118 (10)	121 (12)	0.338	116 (6)	111 (10)	0.317
DBP	76 (8)	77 (11)	0.822	69 (14)	66 (6)	0.620	78 (9)	76 (10)	0.221	68 (9)	66 (8)	0.717
HDL-C	35 (6)	54 (8)	<0.001	64 (19)	79 (14)	0.032	38 (18)	63 (19)	<0.001	55 (27)	68 (19)	0.024
TG	115 (54)	146 (96)	0.223	68 (17)	71 (14)	0.721	113 (63)	107 (56)	0.760	69 (33)	72 (23)	0.784
Glucose	101 (9)	88 (6)	0.011	83 (14)	80 (10)	0.685	102 (8)	82 (21)	0.036	83 (12)	88 (19)	0.472

PPA: postphysical activity; WC: waist circumference; SBP: systolic blood pressure (mmHg); DBP: diastolic blood pressure (mmHg); TG: triglycerides (mg/dL); HDL-C: high-density cholesterol (mg/dL); glucose (mg/dL). ^∗^Paired *t*-test.

**Table 3:** Baseline markers of inflammation, endothelial dysfunction, and oxidative stress in adolescents.

	Women (*n* = 18)			Men (*n* = 20)		
	Mean (SD)			Mean (SD)		
	With MetS	Without MetS	P*	With MetS	Without MetS	P*
Markers of inflammation						
hsCRP	0.16 (0.13)	0.07 (0.04)	0.100	0.09 (0.06)	0.13 (0.08)	0.244
Fg	457 (138)	377 (163)	0.326	438 (177)	417 (141)	0.793
Markers of endothelial cell dysfunction						
PAI-1	37.2 (11)	27.4 (8.9)	0.102	39.2 (5.7)	38.9 (10)	0.937
tPA	5.8 (2.9)	3.2 (1.3)	0.066	4.4 (1.8)	4.9 (2.4)	0.573
vWF	144 (27)	129 (28)	0.308	146 (26)	150 (37)	0.753
FVIII	121 (24)	112 (32)	0.560	112 (27)	134 (42)	0.203
sVCAM-1	17.4 (6.1)	22.8 (14)	0.307	26.9 (17)	19.9 (5.6)	0.374
P-selectin	24.4 (4.7)	20.0 (5.3)	0.119	22.4 (10)	24.0 (8.9)	0.748
Markers of oxidative stress						
Lpox	518 (119)	663 (168)	0.083	605 (223)	589 (269)	0.893
SOD	341 (237)	876 (399)	0.008	681 (385)	510 (709)	0.530
Cat	16.8 (13)	19.9 (16)	0.699	15.4 (14)	13.4 (8.6)	0.746
Gpox	964 (1282)	513 (678)	0.451	2303 (2225)	3792 (3869)	0.328
SOD/Gpox + Cat	17.8 (13)	24.0 (18)	0.475	16.1 (14)	15.1 (11)	0.874

hsCRP: high-sensitivity C-reactive protein (mg/dL); Fg: fibrinogen (mg/dL); PAI-1: plasminogen activator inhibitor type 1 (ng/mL); tPA: tissue plasminogen activator (ng/mL); vWF: von Willebrand factor (U/mL); FVIII: factor VIII (%); sVCAM-1: soluble vascular cell adhesion molecule-1 (U/mL); P-selectin (U/mL); Lpox: lipoperoxidation (nMol MDA/gHb); SOD: superoxide dismutase (*μ*Mol adrenalin/gHb); Cat: catalase (*μ*Mol H_2_O_2_/gHb); Gpox: glutathione peroxidase (*μ*Mol NADPH/gHb). ^∗^Sudent's *t*-test.

**Table 4:** Effects of PA on markers of inflammation, endothelial dysfunction, and stress oxidative in adolescents.

	Women (*n* = 18) (mean ± SD)						Men (*n* = 20) (mean ± SD)					
	With MetS			Without MetS			With MetS			Without MetS		
	Basal	PPA	P*	Basal	PPA	P*	Basal	PPA	P*	Basal	PPA	P*
Markers of inflammation												
hsCRP	0.16 (0.14)	0.13 (0.14)	0.618	0.07 (0.04)	0.05 (0.04)	0.467	0.09 (0.06)	0.12 (0.10)	0.393	0.13 (0.08)	0.11 (0.09)	0.572
Fg	457 (138)	256 (78)	0.009	377 (163)	361 (113)	0.876	438 (178)	267 (55)	0.017	418 (141)	307 (52)	0.050
FVIII	121 (24)	96 (14)	0.016	112 (32)	83 (26)	0.127	112 (27)	104 (19)	0.048	137 (43)	91 (19)	0.045
Markers of endothelial cell damage												
PAI-1	37.2 (11.6)	22.2 (9.5)	0.042	27.3 (8.9)	22.7 (4.9)	0.307	38.9 (5.9)	20.8 (8.4)	0.002	38.9 (10)	25.9 (8.6)	0.041
tPA	5.8 (2.9)	8.6 (3.3)	0.038	3.3 (1.3)	5.4 (3.5)	0.107	4.4 (1.9)	7.9 (4.3)	0.013	4.9 (2.4)	9.2 (3.4)	0.054
vWF	144 (27)	139 (35)	0.685	128 (28)	110 (38)	0.225	146 (26)	119 (50)	0.231	151 (37)	129 (37)	0.328
sVCAM-1	17.4 (6.1)	12.1 (2.8)	0.025	22.8 (13.7)	11.7 (2.7)	0.118	26.9 (17.8)	13.1 (3.8)	0.045	19.9 (5.6)	13.0 (2.3)	0.019
P-selectin	24.4 (4.8)	18.4 (9.1)	0.051	20.0 (5.3)	10.0 (3.9)	0.005	22.5 (10)	12.8 (5.4)	0.028	24.1 (8.9)	14.9 (7.2)	0.018
Markers of oxidative stress												
Lpox	518 (119)	371 (50)	0.022	574 (107)	394 (39)	0.089	611 (215)	354 (65)	0.002	448 (144)	388 (42)	0.324
SOD	341 (237)	800 (393)	0.032	778 (276)	681 (484)	0.757	697 (390)	803 (315)	0.487	655 (809)	719 (302)	0.825
Cat	16.8 (13.3)	9.2 (17.9)	0.405	26.1 (17.2)	5.8 (3.2)	0.118	16.5 (16.0)	9.8 (19.6)	0.500	15.4 (9.5)	3.6 (4.1)	0.037
Gpox	964 (1282)	3137 (5713)	0.308	208 (103)	3126 (3549)	0.202	2349 (2519)	3055 (4397)	0.714	4588 (4436)	1333 (722)	0.164
SOD/Gpox + Cat	17.8 (13.5)	10.0 (17.9)	0.387	31.1 (18.5)	6.2 (3.5)	0.087	17.3 (16.1)	10.5 (20.6)	0.496	17.7 (12.7)	4.7 (3.9)	0.064

PPA: postphysical activity; hsCRP: high-sensitivity C-reactive protein (mg/dL); Fg: fibrinogen (mg/dL); PAI-1: plasminogen activator inhibitor type 1 (ng/mL); tPA: tissue plasminogen activator (ng/mL); vWF: von Willebrand factor (UI/mL); FVIII: factor VIII (%); sVCAM-1: soluble vascular cell adhesion molecule-1 (U/mL); P-selectin (U/mL); Lpox: lipoperoxidation (nMol MDA/gHb); SOD: superoxide dismutase (*μ*Mol adrenalin/gHb); Cat: catalase (*μ*Mol H_2_O_2_/gHb); Gpox: glutathione peroxidase (*μ*Mol NADPH/gHb); ^∗^paired *t*-test.
